# Motion planning framework based on dual-agent DDPG method for dual-arm robots guided by human joint angle constraints

**DOI:** 10.3389/fnbot.2024.1362359

**Published:** 2024-02-22

**Authors:** Keyao Liang, Fusheng Zha, Wei Guo, Shengkai Liu, Pengfei Wang, Lining Sun

**Affiliations:** State Key Laboratory of Robotics and System, Harbin Institute of Technology, Harbin, China

**Keywords:** trajectory planning, reinforcement learning, dual-agent depth deterministic strategy gradient, human experience constrains guidance, motion parameter mapping

## Abstract

**Introduction:**

Reinforcement learning has been widely used in robot motion planning. However, for multi-step complex tasks of dual-arm robots, the trajectory planning method based on reinforcement learning still has some problems, such as ample exploration space, long training time, and uncontrollable training process. Based on the dual-agent depth deterministic strategy gradient (DADDPG) algorithm, this study proposes a motion planning framework constrained by the human joint angle, simultaneously realizing the humanization of learning content and learning style. It quickly plans the coordinated trajectory of dual-arm for complex multi-step tasks.

**Methods:**

The proposed framework mainly includes two parts: one is the modeling of human joint angle constraints. The joint angle is calculated from the human arm motion data measured by the inertial measurement unit (IMU) by establishing a human-robot dual-arm kinematic mapping model. Then, the joint angle range constraints are extracted from multiple groups of demonstration data and expressed as inequalities. Second, the segmented reward function is designed. The human joint angle constraint guides the exploratory learning process of the reinforcement learning method in the form of step reward. Therefore, the exploration space is reduced, the training speed is accelerated, and the learning process is controllable to a certain extent.

**Results and discussion:**

The effectiveness of the framework was verified in the gym simulation environment of the Baxter robot's reach-grasp-align task. The results show that in this framework, human experience knowledge has a significant impact on the guidance of learning, and this method can more quickly plan the coordinated trajectory of dual-arm for multi-step tasks.

## 1 Introduction

In recent years, more and more researchers have paid attention to dual-arm robots, which are more similar to human beings in terms of configuration, joint freedom, and working space. They can better replace human tasks by imitating human arms (Wang et al., 2022). Compared with single-arm robots, dual-arm robots have significant advantages in precision assembly with high coordination and multi-object assembly in unstructured environments. Because of the high degree of freedom and high coordination of the dual-arm robot, it requires high dimensions and strong coupling for its motion planning. In particular, for multi-step complex tasks, the motion process of the dual-arm robot can be divided into multiple sub-task processes, which increases the dimension of motion planning from the task planning level. The traditional motion planning method mainly solves dual-arm robots' obstacle avoidance motion planning problem using constraint model establishment and non-linear solutions (Vahrenkamp et al., 2012; Fang et al., 2015; Giftthaler et al., 2017). However, this method has little effect on dual-arm robots' multi-step coordination tasks, which limits the application of dual-arm robots.

With the development of machine learning methods, more and more researchers use intelligent learning methods to complete the motion planning of multi-step coordination tasks of dual-arm robots (Bing et al., 2022a, 2023b,d). Learning-based motion planning methods are mainly divided into imitation learning and reinforcement learning. The motion planning method based on imitation learning learns the motion features from the teaching demonstration and then reproduces the demonstration task on the robot. Maeda et al. (2020) proposed a demonstration programming method that automatically derives task constraints from data for constraint-based robot controllers using the Dirichlet Process Gaussian Mixture Model (DPGMM) and Gaussian Mixture Regression (GMR) method. In the study by Mronga and Kirchner (2021), phase portrait movement primitives (PPMPs), which can predict the dynamics of the low-dimensional phase space and then can be used to control the high-dimensional kinematics of the task, were proposed. In the study by Dong et al. (2022), a model-based learnable graph attention network (GAT) was used to learn task-level skills from human demonstration passively. It was validated in a humanoid robot task experiment of waving and grasping boxes. This category method can realize human imitation from the level of learning content and effectively learn human motion knowledge, but it can not optimize or learn new trajectories independently.

The motion planning method based on reinforcement learning enables the agent to explore learning motion strategies by interacting with the environment (Bing et al., 2022b, 2023a; Chu et al., 2022). For example, Ren and Ben-Tzvi (2020) proposed an advising reinforcement learning approach based on the depth deterministic strategy gradient (DDPG) and hindsight experience replay (HER), which applies the teacher-student framework to a continuous control environment with sparse rewards to solve the problem of extended agents. In the study by Jiang et al. (2021), a multiagent twin delayed deep deterministic policy gradient (MATD3) algorithm was proposed for the on-orbit acquisition mission of a space robot arm to generate a real-time inverse kinematics solution for the coordinated robot arm. In the study by Tang et al. (2022), the proximal policy optimization (PPO) algorithm with continuous rewards was used for trajectory planning of the two-arm robot, and the reward and punishment function was designed based on the artificial potential field (APF) method so that the dual-arm robot could approach and support patients in a complex environment. This category method imitates human beings from the level of learning style and mimics the human trial and error reward learning mechanism. However, the high-dimensional problem of trajectory planning brought by dual-arm multi-step tasks which will make the search space of reinforcement learning larger, the training process easily falls into local optimal, and the training results are difficult to converge.

In the previous study, the DADDPG algorithm proposed could reduce and decouple the dual-arm trajectory planning problem to a certain extent and successfully plan the dual-arm coordination trajectory for multi-objective tasks (Liang et al., 2023). Based on the DADDPG algorithm, this study proposes a motion planning framework guided by the human joint angle constraints, which simultaneously realizes the human-like learning content and learning style. By introducing human joint angle constraint, this method reduces the exploration space of reinforcement learning, rationalizes its exploration, makes its learning controllable to a certain extent, and speeds up the learning speed.

Building a robot's structure or control algorithm by imitating humans or animals has long been one of the potential means of improving robot performance (Bing et al., 2023c). The bionics-based human-like arm motion planning method extracts biomarkers and rules from recorded movements for simulating arm motion trajectory (Gulletta et al., 2020). For example, Kim et al. proposed a method to extract human arm movement features from the motion capture database, characterize human arm movement according to elbow elevation angle, and use this representation to generate human-like movements in real-time (Kim et al., 2006; Shin and Kim, 2014). In the study by Suárez et al. (2015), a motion planning method for a dual-arm anthropomorphic system was proposed, and a new basis vector of the dual-arm configuration space returned by principal component analysis (PCA) was used to characterize the dual-arm synergy. In this study, the human joint angle is calculated from the demonstration data collected by IMU and mapped to the robot model. Then, the joint angle constraint is extracted piecewise from the multi-group human demonstration and used to guide the autonomous learning of the multi-step coordination trajectory of dual-arm robots.

Three existing learning optimization methods use empirical knowledge to guide reinforcement learning: reward function optimization, exploration behavior optimization, and network parameter initialization (Taylor et al., 2011; Bougie et al., 2018; Xiang and Su, 2019). Among them, optimizing reward function is the most consistent with human behavior patterns. It models the reward function of the reinforcement learning method based on the empirical knowledge model, which can guide reinforcement learning intuitively and effectively (Tian et al., 2021). The segmented guided step reward of this study is designed to make the joint angle constraints guide the DADDPG method to quickly learn the dual-arm coordination trajectory for complex multi-step tasks.

## 2 Methodology

**Figure 1:**
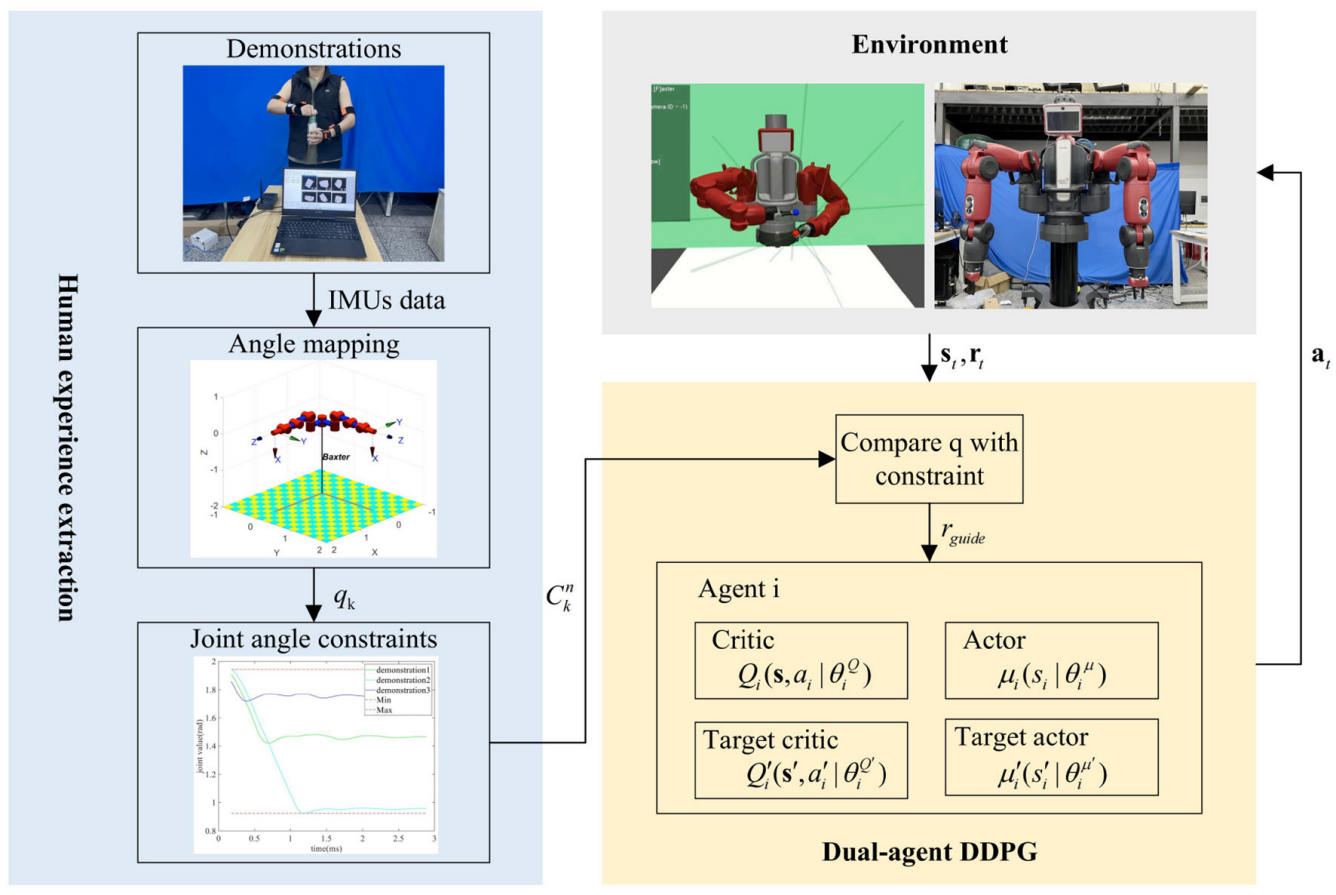
The proposed framework block diagram.

This study proposes a motion planning framework for dual-arm robots guided by human joint constraints based on the DADDPG method, as shown in Figure 1. In the proposed framework, the joint angle is calculated from the demonstration data collected by IMU and mapped to the robot model. Then, the joint angle constraint is extracted piecewise from multiple groups of human demonstration. The joint angle constraint is then used to guide the DADDPG method to quickly learn the dual-arm coordination trajectory for complex multi-step tasks through reward distribution. The following is a detailed introduction from four aspects: joint mapping model, joint angle constraint, reinforcement learning method, and reward guidance design.

### 2.1 Human-robot joint mapping model

**Figure 2:**
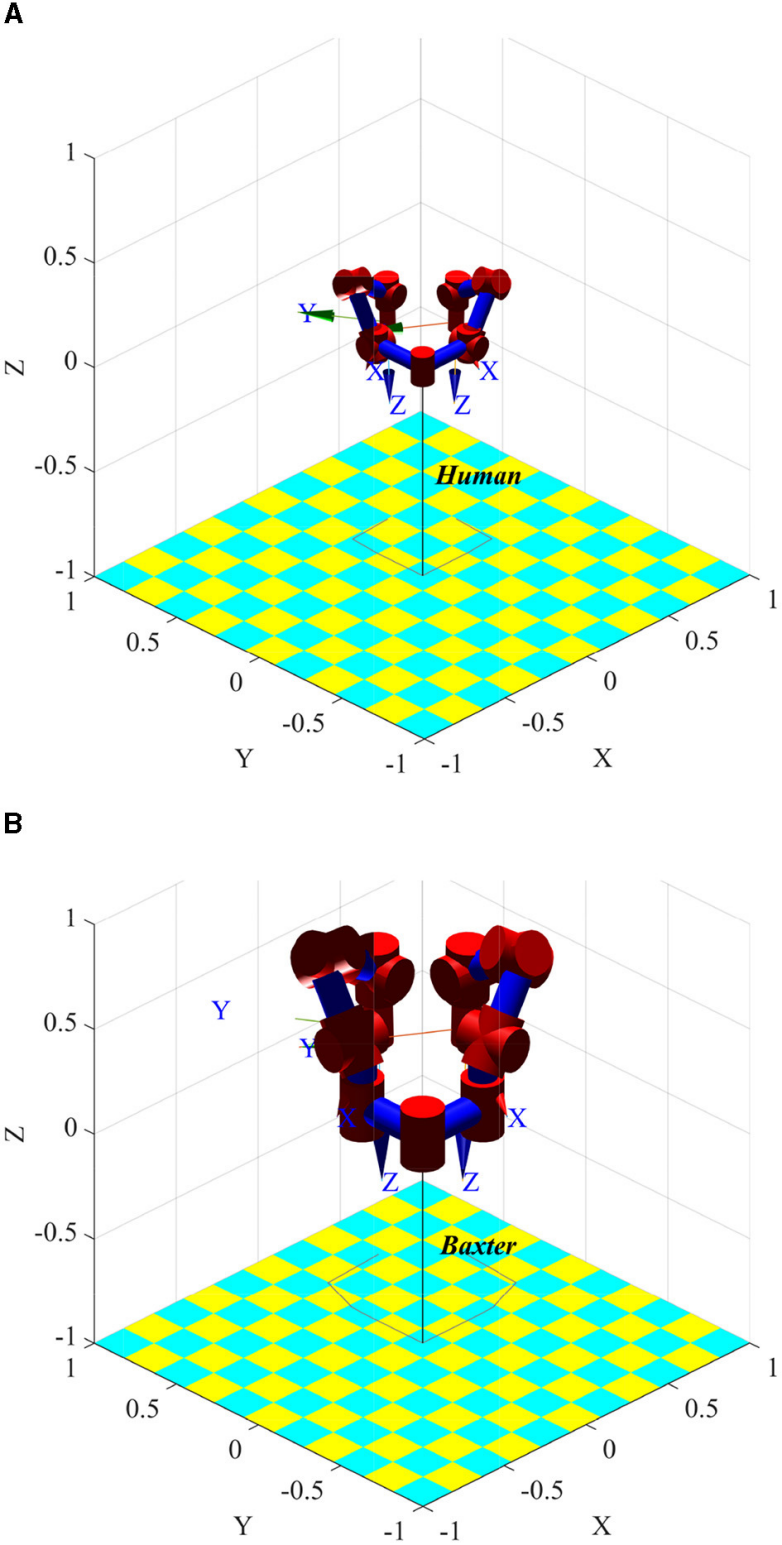
Comparison of human and robot pose when joint angle *ql* = *qr* = [−0.078, −0.968, −1.150, 1.923, 0.648, 1.008, −0.483] is set. **(A)** Simplified kinematic model of human arms. **(B)** Kinematic model of Baxter robot.

The human arm has three joints: shoulder, elbow, and wrist. Among them, the shoulder joint has three rotational degrees of freedom, the elbow joint has two rotational degrees of freedom, and the wrist joint has two rotational degrees of freedom. The kinematics model of the human arm and Baxter is established using the standard D-H method (Denavit and Hartenberg, 1955). The parameters are shown in Tables 1, 2. *q*_1_, *q*_2_, *q*_3_ is the rotation angle corresponding to the shoulder joint, *q*_4_, *q*_5_ is the rotation angle corresponding to the elbow joint, and *q*_6_, *q*_7_ is the rotation angle corresponding to the wrist joint. Input the same joint angle value, and the posture of the two models is consistent, as shown in Figure 2. Figure 2A is the simplified kinematics model of the human arms, and Figure 2B is the kinematics model of the Baxter robot.

**Table 1:** D-H parameters of the human arm(right).

**Item**	**θ_*i*_**	***d*_*i*_**	***a*_*i*_**	**α_*i*_**	***offset*_*i*_**
1	*q*_1_	0	0	$\frac{\pi }{2}$	π
2	*q*_2_	0	0	$\frac{\pi }{2}$	$-\frac{\pi }{2}$
3	*q*_3_	*l*_1_	0	$\frac{\pi }{2}$	π
4	*q*_4_	0	0	$\frac{\pi }{2}$	π
5	*q*_5_	*l*_2_	0	$\frac{\pi }{2}$	π
6	*q*_6_	0	0	$\frac{\pi }{2}$	π
7	*q*_7_	*l*_3_	0	0	0

*l*_1_ is the distance from the shoulder to the elbow, *l*_2_ is the distance from the elbow to the wrist, and *l*_3_ is the distance from the wrist to the palm.

**Table 2:** D-H parameters of Baxter arm(right).

**Item**	**θ_*i*_**	***d*_*i*_**	***a*_*i*_**	**α_*i*_**	***offset*_*i*_**
1	*q*_1_	0.27	0.069	$-\frac{\pi }{2}$	0
2	*q*_2_	0	0	$\frac{\pi }{2}$	$\frac{\pi }{2}$
3	*q*_3_	0.364	0.069	$-\frac{\pi }{2}$	0
4	*q*_4_	0	0	$\frac{\pi }{2}$	0
5	*q*_5_	0.375	0.01	$-\frac{\pi }{2}$	0
6	*q*_6_	0	0	$\frac{\pi }{2}$	0
7	*q*_7_	0.28	0	0	0

**Figure 3:**
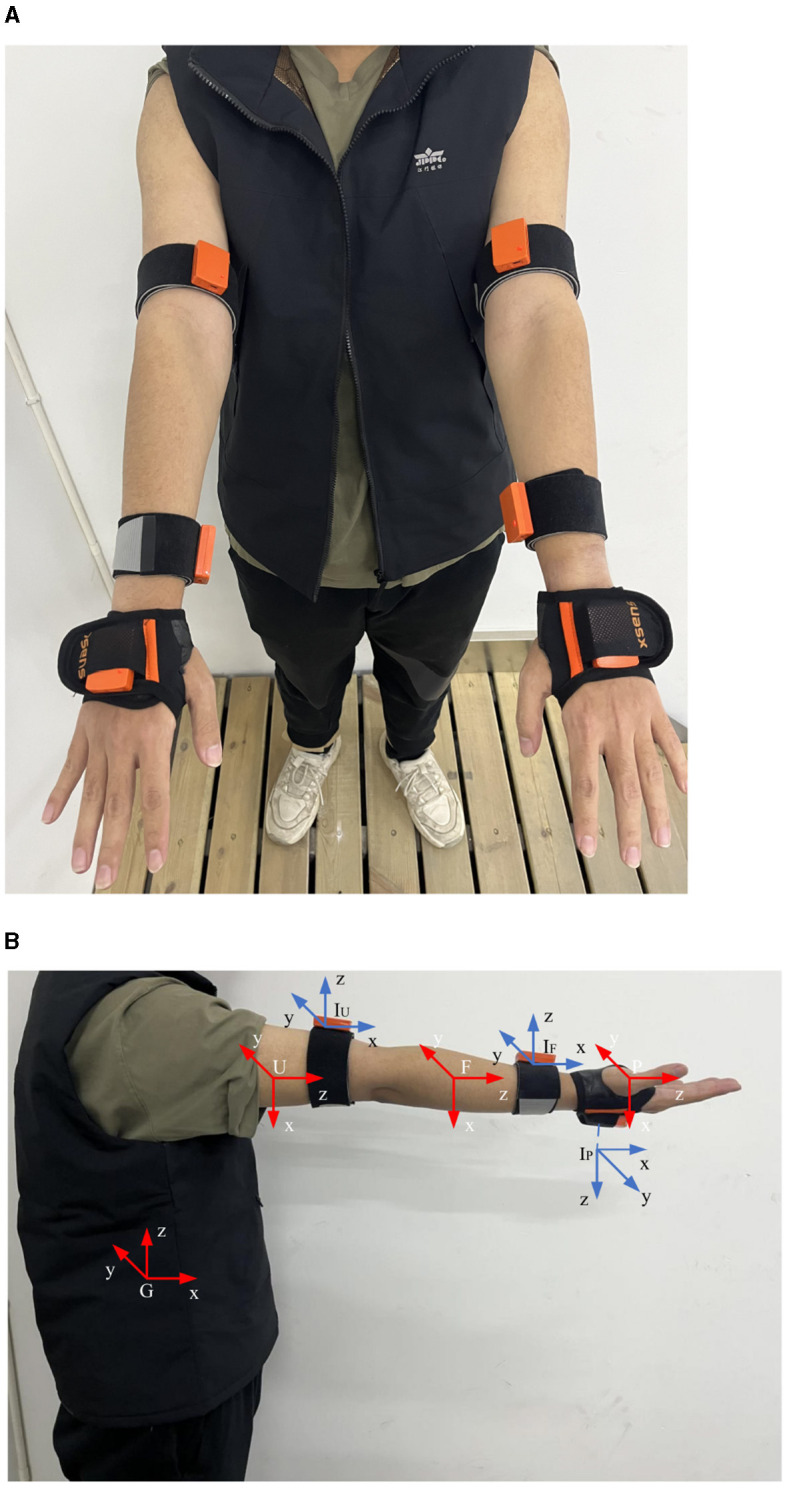
IMUs and coordinate frame schematic. **(A)** The position of the IMU on the presenter's arm. **(B)** Coordinate frame diagram.

The following describes how to solve the corresponding joint angle from the demonstration data measured by the IMU. The presenter wears six IMUs, as shown in Figure 3A, three for each arm, to measure the spatial orientation of the upper arm, forearm, and palm. Taking the right arm as an example, the coordinate system is presented in Figure 3B: *G* is the global coordinate frame, *U* is the upper arm coordinate frame, *F* is the forearm coordinate frame, *P* is the palm coordinate frame, *I*_*U*_ is the coordinate frame of the IMU on the upper arm, *I*_*F*_ is the coordinate frame of the IMU on the forearm, and *I*_*P*_ is the coordinate frame of the IMU on the palm.

The initial position of the right arm joint angle is specified as *qr*_0_ = [*q*_1_, *q*_2_, *q*_3_, *q*_4_, *q*_5_, *q*_6_, *q*_7_] = [0, 0, 0, 0, 0, 0, 0]. In the initial joint angle configuration, the orientation of each frame of the right arm with respect to the global frame is represented as: $R_{GU}^{0}=R_{GF}^{0}=R_{GP}^{0}=\left[0,0,1;0,1,0;-1,0,0\right]$. The measured values of the IMU on the upper arm, forearm, and palm are $R_{GI_{U}}^{0}$, $R_{GI_{F}}^{0}$, and $R_{GI_{P}}^{0}$, respectively, that is, the orientation of the IMU's frame with respect to the global frame. The orientation of the IMU relative to the arm can be determined as shown in Equation (1):


(1) $$\{\begin{array}{c} R_{UI_{U}}={\left(R_{GU}^{0}\right)}^{T}R_{GI_{U}}^{0}\\ R_{FI_{F}}={\left(R_{GF}^{0}\right)}^{T}R_{GI_{F}}^{0}\\ R_{PI_{P}}={\left(R_{GP}^{0}\right)}^{T}R_{GI_{P}}^{0} \end{array}$$

where *R*_*UI*_*U*__ is the orientation of the IMU's frame on the upper arm relative to the upper arm's frame, *R*_*FI*_*F*__ is the orientation of the IMU's frame on the forearm relative to the forearm's frame, and *R*_*PI*_*P*__ is the orientation of the IMU's frame on the palm relative to the palm's frame.

When the arm moves to a new position, the orientation of the IMU concerning the arm remains unchanged, assuming that the new orientations of the IMU's frame relative to the global frame are $R_{GI_{U}}^{new}$, $R_{GI_{F}}^{new}$, and $R_{GI_{P}}^{new}$. The orientation of the upper arm, forearm, and palm can be calculated using Equation (2):


(2) $$\{\begin{array}{c} R_{GU}^{new}=R_{GI_{U}}^{new}{\left(R_{UI_{U}}\right)}^{T}\\ R_{GF}^{new}=R_{GI_{F}}^{new}{\left(R_{FI_{F}}\right)}^{T}\\ R_{GP}^{new}=R_{GI_{P}}^{new}{\left(R_{PI_{P}}\right)}^{T} \end{array}$$

where $R_{GU}^{new}$ is the orientation of the upper arm's frame with respect to the global frame in new position, $R_{GF}^{new}$ is the orientation of the forearm's frame with respect to the global frame in new position, and $R_{GP}^{new}$ is the orientation of the palm's frame with respect to the global frame in new position. The relationship between the orientation of the upper arm's frame with respect to the global frame and the joint angles *q*_1_, *q*_2_, and *q*_3_ is shown in Equation (3):


(3) $$\begin{array}{l} R_{GU}^{new}=R_{X}\left(-q_{1}\right)R_{Y}\left(q_{2}\right)R_{Z}\left(q_{3}\right) \end{array}$$

By substituting Equation (2) into Equation (3), the joint angles *q*_1_, *q*_2_, and *q*_3_ corresponding to the shoulder joint can be calculated as Equation (4):


(4) $$\{\begin{array}{c} q_{1}=-atan2\left(-R_{GU}^{new}\left(2,3\right),R_{GU}^{new}\left(3,3\right)\right)\\ q_{3}=atan2\left(-R_{GU}^{new}\left(1,2\right),R_{GU}^{new}\left(1,1\right)\right)\\ q_{2}=atan2\left(-R_{GU}^{new}\left(1,3\right),\frac{R_{GU}^{new}\left(1,1\right)}{cos(q_{3})}\right) \end{array}$$

The orientation of the forearm relative to the upper arm can be calculated using Equation (5):


(5) $$\begin{array}{l} R_{UF}^{new}={\left(R_{GU}^{new}\right)}^{T}R_{GF}^{new} \end{array}$$

The relationship between the orientation of the forearm's frame with respect to the global frame and the joint angles *q*_4_ and *q*_5_ is shown in Equation (6):


(6) $$\begin{array}{l} R_{UF}^{new}=R_{Y}\left(q_{4}\right)R_{Z}\left(q_{5}\right) \end{array}$$

By substituting Equation (5) into Equation (6), the joint angles *q*_4_ and *q*_5_ corresponding to the elbow joint can be calculated as Equation (7):


(7) $$\{\begin{array}{c} q_{4}=atan2\left(-R_{UF}^{new}\left(1,3\right),R_{UF}^{new}\left(3,3\right)\right)\\ q_{5}=atan2\left(-R_{UF}^{new}\left(2,1\right),R_{UF}^{new}\left(2,2\right)\right) \end{array}$$

The orientation of the palm relative to the forearm can be calculated using Equation (8):


(8) $$\begin{array}{l} R_{FP}^{new}={\left(R_{GF}^{new}\right)}^{T}R_{GP}^{new} \end{array}$$

The relationship between the orientation of the palm's frame with respect to the global frame and the joint angles *q*_6_ and *q*_7_ is shown in Equation (9):


(9) $$\begin{array}{l} R_{FP}^{new}=R_{Y}\left(q_{6}\right)R_{Z}\left(q_{7}\right) \end{array}$$

By substituting Equation (8) into Equation (9), the joint angles *q*_6_ and *q*_7_ corresponding to the wrist joint can be calculated as Equation (10):


(10) $$\{\begin{array}{c} q_{6}=atan2\left(-R_{FP}^{new}\left(1,3\right),R_{FP}^{new}\left(3,3\right)\right)\\ q_{7}=atan2\left(-R_{FP}^{new}\left(2,1\right),R_{FP}^{new}\left(2,2\right)\right) \end{array}$$

In this way, the right arm joint angle is calculated, and the left arm joint angle can also be calculated by the above method.

### 2.2 Joint angle constraint

A human demonstration can be divided into multiple trajectories for a complex multi-step task. For example, a bottle cap screwing task can be broken down into reaching, grabbing, aligning, and screwing steps. Suppose a multi-step task is artificially divided into *N* sub-tasks; in the sub-task *n*, the angle constraint of the *k*-th joint of the human arm can be expressed as Equation (11):


(11) $$\begin{array}{l} a_{k}^{n}\leq q_{k}^{n}\leq b_{k}^{n} \end{array}$$

where 0 < *n* ≤ *N*, 0 < *k* ≤ 14, $a_{k}^{n}$ is the lower limit for the *k*-th joint angle in sub-task *n*, $b_{k}^{n}$ is the upper limit for the *k*-th joint angle in sub-task *n*.

When there are *M* groups of human demonstrations, the trajectories can be divided into M*N sub-trajectories, where *N* is the number of sub-tasks. Then, for sub-task *n*, the angle constraint of the *k*-th human arm joint can be expressed as Equation (12):


(12) $$C_{k}^{n}:\;\{\begin{array}{c} q_{k}^{n}\geq \min\left(a_{k,1}^{n},a_{k,2}^{n},\cdots ,a_{k,m}^{n}\right)\\ q_{k}^{n}\leq \max\left(b_{k,1}^{n},b_{k,2}^{n},\cdots ,b_{k,m}^{n}\right) \end{array}$$

where 0 < *m* ≤ *M*, $a_{k,m}^{n}$ is the lower limit of the *k*-th joint angle in the sub-task *n* of the demonstration *m*, $b_{k,m}^{n}$ is the upper limit of the *k*-th joint angle in the sub-task *n* of the demonstration *m*.

### 2.3 Reinforcement learning method

The DADDPG method proposed in previous study can plan the coordinated trajectories of dual-arm robots for multi-objective tasks (Liang et al., 2023). In this study, the DADDPG algorithm is chosen as the algorithm of reinforcement learning, which uses two agents to plan the coordinated trajectory of the left arm and the right arm simultaneously. Each agent contains four networks: Actor $\mu_{i}\left(s_{i}|{\theta_{i}}^{\mu }\right)$, Critic $Q_{i}\left(s,a_{i}|{\theta_{i}}^{Q}\right)$, Target Actor $\mu_{i}^{\prime }\left(s_{i}^{\prime }|\theta_{i}^{\mu^{\prime }}\right)$, and Target Critic $Q_{i}^{\prime }\left(s^{\prime },a_{i}^{\prime }|\theta_{i}^{Q^{\prime }}\right)$, where *i* = 1, 2.

For the agent *i*, the parameters of the Critic network are updated by minimizing MSBE loss *Lc* by the gradient descent method using Equation (13) (Liang et al., 2023):


(13) $$\begin{array}{l} L_{c}={\left(y_{i}-q_{i}\right)}^{2}=(Q_{i}(s_{j},a_{j,i}|\theta_{i}^{Q})-r_{j,i}+\gamma (1-done)\\ Q^{\prime }{\;}_{i}(s_{j+1},\mu^{\prime }{\;}_{i}(s_{j+1,i}|\theta_{i}^{\mu^{\prime }})|\theta_{i}^{Q^{\prime }}){)}^{2} \end{array}$$

The parameters of the Actor network are updated by maximizing the cumulative expected return *J* of agent *i* by the gradient ascent method using Equation (14) (Liang et al., 2023):


(14) $$\begin{array}{l} {▽}_{\theta_{i}^{\mu }}J=\underset{\text{s}\sim \mu_{1},\mu_{2}}{E}\left[{▽}_{a_{i}}Q_{i}\left(\text{s},a_{i}|\theta^{Q}\right){|}_{\text{s}={\text{s}}_{j},a_{i}=\mu_{i}\left(s_{j,i}\right)}{▽}_{\theta_{i}^{\mu }}\mu_{i}\left(s_{i}|\theta_{i}^{\mu }\right)|s_{j,i}\right] \end{array}$$

The parameters of the target networks are updated by way of soft update using Equation (15) (Liang et al., 2023):


(15) $$\{\begin{array}{c} \theta_{i}^{Q^{\prime }}\leftarrow \tau \theta_{i}^{Q}+(1-\tau )\theta_{i}^{Q^{\prime }}\\ \theta_{i}^{\mu^{\prime }}\leftarrow \tau \theta_{i}^{\mu }+(1-\tau )\theta_{i}^{\mu^{\prime }} \end{array}$$

### 2.4 Guided reward design

Based on the reward function designed in the previous study (Liang et al., 2023), this study develops the segmented guided step reward term so that the human joint angle constraint can guide the learning process of the reinforcement learning method and narrow its exploration space. The reward of agent *i* can be calculated using the reward function. The segmented guided step reward term *r*_*guide*_ is shown in Equation (16):


(16) $$\begin{array}{l} r_{guide}=\underset{k}{\sum }r_{n,k} \end{array}$$

where $n=\{\begin{array}{ll} 1 & \text{ if }goal_{1}=False\\ 2 & \text{ if }goal_{1}=True,goal_{2}=False\\ \cdots & \;\\ N & \text{ if }goal_{1},goal_{2},\cdots ,goal_{N-1}=True,goal_{N}=False \end{array}$, *goal*_*n*_ is the target of sub-task *n*. $r_{n,k}=\{\begin{array}{ll} c_{0} & \text{ if }q_{k}\text{ satisfies the constraint }C_{k}^{n}\\ -c_{0} & \text{ else } \end{array}$, *c*_0_ is a positive constant.

The step reward term of the reward function is shown as Equation (17):


(17) $$\begin{array}{l} r_{step}=-distance\left(pos\text{\_}gripper_{i},pos\text{\_}finalgoal_{i}\right)+r_{guide} \end{array}$$

The reward function is the sum of the three items shown in Equation (18) (Liang et al., 2023):


(18) $$\begin{array}{l} R=r_{step}+r_{goal}+r_{coordinate} \end{array}$$

## 3 Experiment

### 3.1 Validation of joint angle mapping method

**Figure 4:**
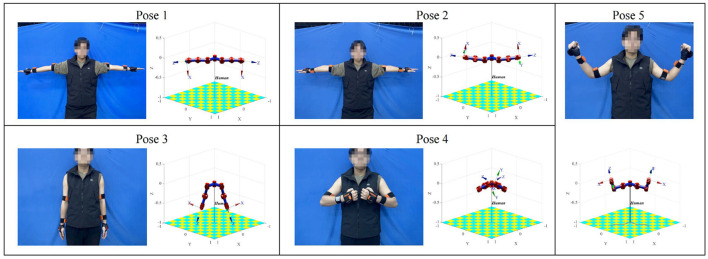
Comparison of human arm sampling pose and D-H visual model pose.

This experiment set up a human arm movement trajectory, and the arm posture data were measured using IMUs. The arm joint angle was calculated using the method in Section 2.1 and input into a simplified D-H model of the human arm. The human arm pose sampled at five positions was compared with the D-H visualization model pose, as shown in Figure 4.

In the D-H visualization model, the X-axis of the coordinate frame at the end of the right arm corresponds to the direction of the right hand, and the Z-axis corresponds to the direction of the fingers when the right hand is opened. The X-axis of the coordinate frame at the end of the left arm corresponds to the direction of the left hand, and the Z-axis corresponds to the direction of the fingers when the left hand is opened. As can be observed from the comparison in Figure 4, the orientation of the two end coordinate frames of the model is consistent with the orientation of the IMUs worn on the demonstrator's palms, and the posture of the demonstrator's arm and the model's arm is also very similar. Therefore, the joint angle calculation and mapping method in this study are effective.

### 3.2 Validation of joint angle constraint guidance method

In this section, the effectiveness of the proposed joint angle constraint guidance method was verified on a Baxter robot in a GYM simulation environment for multi-step tasks. The set multi-step task consists of three sub-steps: reach, grasp, and align. Ten groups of demonstration data of human execution of the reach-grasp-align task were collected, calculated, and processed to obtain joint angle constraints. Then, the constraint guidance is added to each time step of DADDPG algorithm learning trajectory. To verify the framework's effectiveness proposed in this study, the learning effects of single-segment constraint introduction, multi-segment constraint introduction, and unconstrained introduction are compared.

#### 3.2.1 Experiment settings

**Figure 5:**
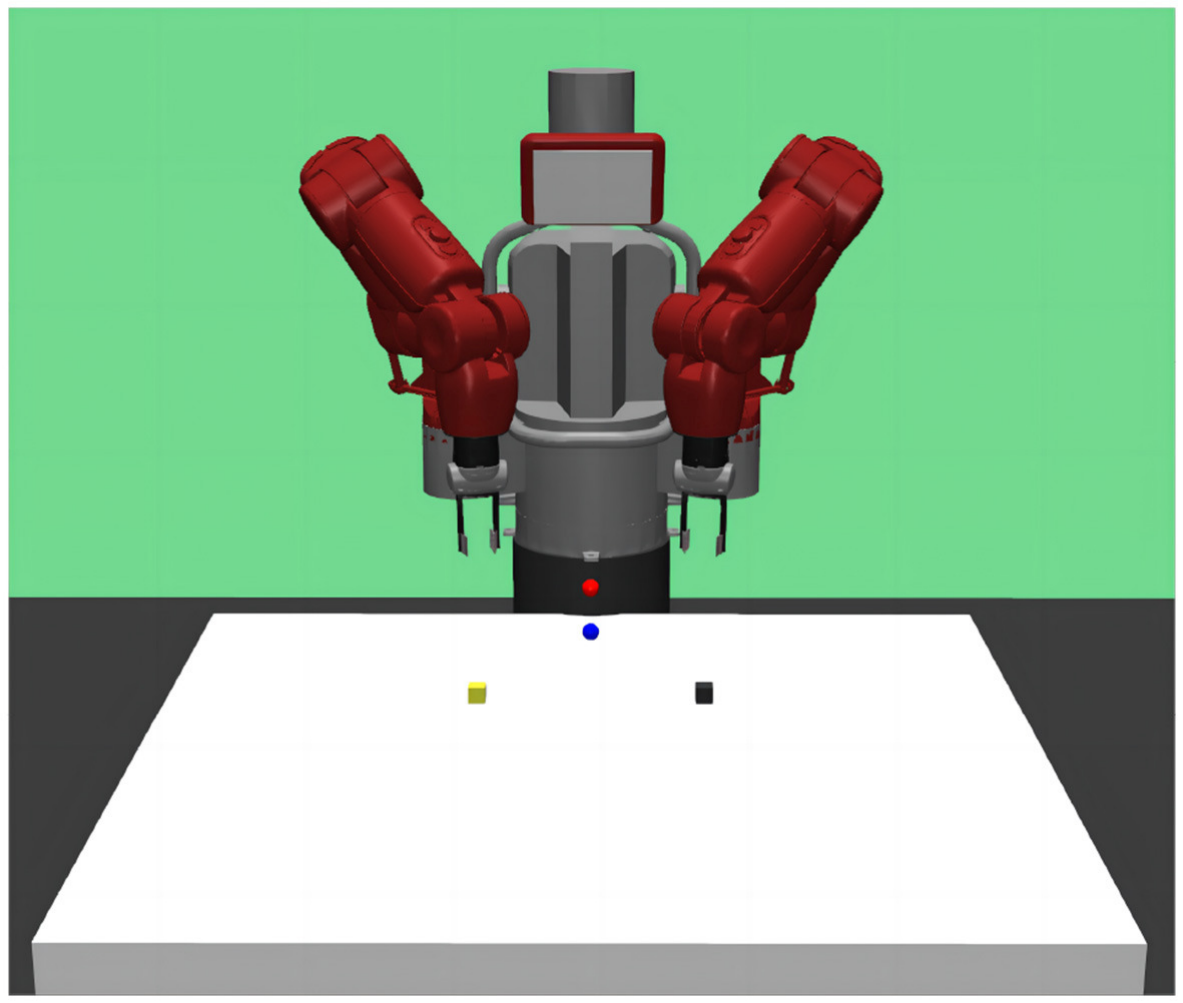
GYM simulation environment for Baxter robot's reach-grasp-align task. Baxter's arms reach the position of their respective object box, then grasp their respective object box, and finally align the two object boxes.

The reach-grasp-align task scene of the Baxter robot in the GYM simulation environment is shown in Figure 5. The black block is the operating object of the left arm, the yellow block is the operating object of the right arm, the red sphere is the target position of the left arm, and the blue sphere is the target position of the right arm. The parameters of the DADDPG algorithm were set the same as in the study by Liang et al. (2023), except that the dimension of observation was changed.

##### 3.2.1.1 Constraints

This experiment used two types of constraints: single-segment and multi-segment constraints. When the calculated joint angle data were not segmented, the entire motion process included reach, grasp, and align sub-steps, and a single segment constraint *C*1 was obtained. When the calculated joint angle data were segmented, it was divided into reach, grasp, and align sub-steps. Because the joint movement of the grasp substep was tiny, constraint *C*2 was obtained from the joint angle data of the reach substep and the grasp substep, and constraint *C*3 was obtained from the joint angle data of the align substep. *C*2 and *C*3 form a multi-segment constraint.

##### 3.2.1.2 Observation

Joint angle constraint guidance must introduce the state of the robot's joint angle in the observation to guide the agent's learning. The observation was therefore set as: **s** = (*s*_*l*_, *s*_*r*_), *s*_*l*_ is the state of the robot's left arm and its target, including the position of the left gripper, the position of the left arm joint angle, the position of the left object, the relative position of the left object and the left gripper, the state of the two fingers of the left gripper, the orientation of the left object, the linear velocity of the left object, the angular velocity of the left object, the linear velocity of the left gripper, the speed of the two fingers of the left gripper, and the position of the left target. *s*_*r*_ is defined as the variable corresponding to the right arm.

When no constraints are introduced, the state of the robot's joint angle is not required. The observation was the same as in the study by Liang et al. (2023).

##### 3.2.1.3 Reward

This experiment used the reward function designed in Section 2.4. Set the guiding reward constant to 0.01 for the reach and grasp stages and 0.05 for the align stage. Set the reward of subgoal reaching *rg*_1_ = 1, the reward of subgoal grasping *rg*_2_ = 20, the reward of coordinate *rc* = 4000.

#### 3.2.2 Results

**Figure 6:**
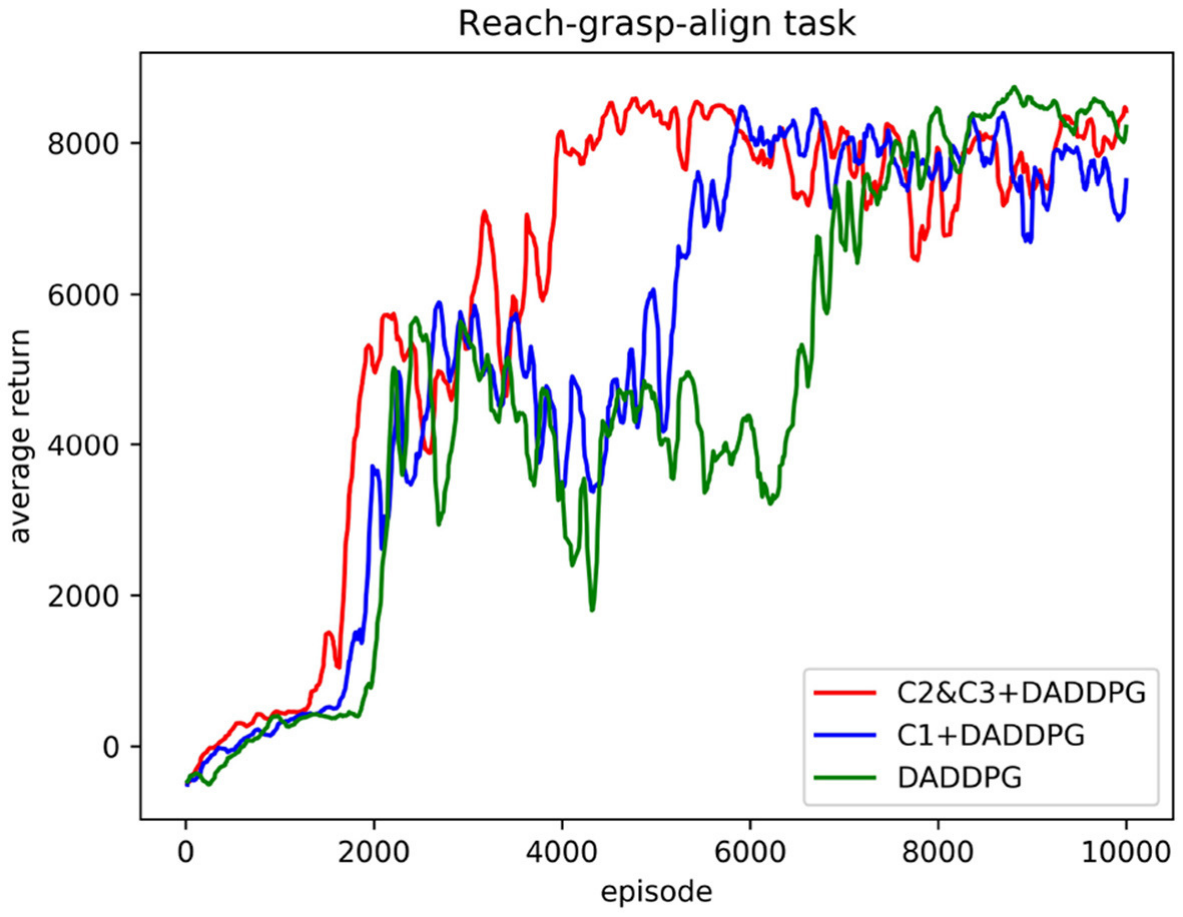
Comparison of the average cumulative return curves of the DADDPG algorithm with single-segment constraint introduction, multi-segment constraint introduction, and no constraint introduction trained in the reach-grasp-align task of the dual-arm robot.

**Figure 7:**
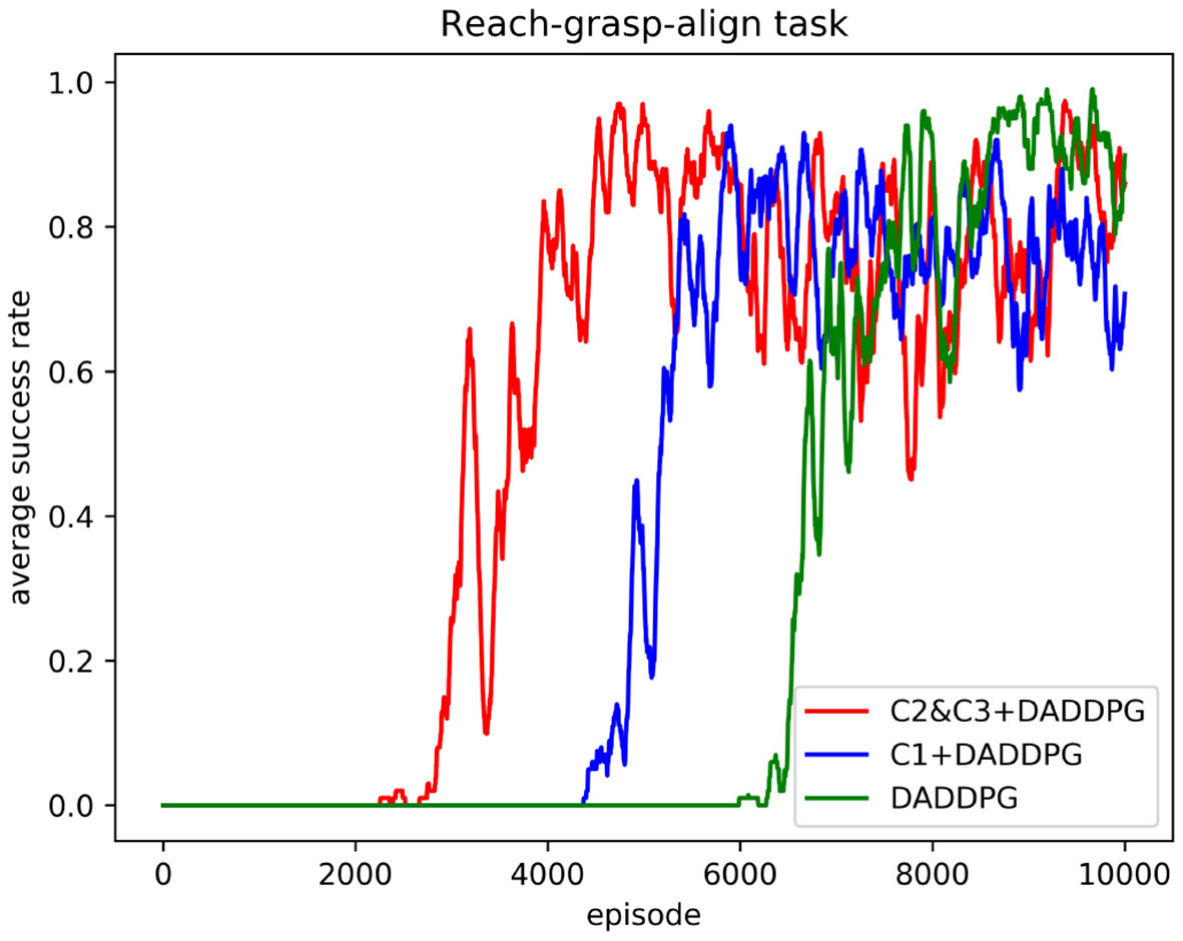
Comparison of average success rate curves of the DADDPG algorithm with single-segment constraint introduction, multi-segment constraint introduction, and no constraint introduction in the reach-grasp-align task of the dual-arm robot.

The comparison of training curves of the DADDPG algorithm with single-segment constraint introduction, multi-segment constraint introduction, and no constraint introduction in the reach-grasp-align task of the dual-arm robot is shown in Figures 6, 7.

Figure 6 shows the curve comparison of the average cumulative return as the number of training increases. The DADDPG algorithm with single-segment constraint introduction, multi-segment constraint introduction, and no constraint introduction was trained 10,000 episodes. As can be observed from the figure, the average cumulative return curve of an agent guided by multi-segment constraints *C*2&*C*3 converges at approximately 4,000 episodes, that of an agent guided by single-segment constraints *C*1 converges at approximately 5,600 episodes, and that of an agent guided by no constraints converges at approximately 7,000 episodes. Regarding the number of training episodes for the average cumulative return curve convergence, the number of training episodes required for converging multi-segment constrained guided agents is 71% for single-segment constrained guided agents and 57% for unconstrained guided agents. The number of necessary training episodes for single-segment constrained guided agent convergence is 80% of that needed for unconstrained guided agents. The results show that adding single-segment constraints to DADDPG can significantly improve the speed of training convergence. Improving the rate of training convergence by introducing constraints in segments is more prominent. This verifies the validity of the motion planning framework for two-arm robots based on the DADDPG method, which is guided by human joint constraints.

Figure 7 shows the curve comparison of the average success rate as the number of training increases. As can be observed from the figure, the average success rate of the agent guided by multi-segment constraint *C*2&*C*3 is 0.85 or above approximately 4,480 episodes, and the average success rate of the agent guided by single-segment constraint *C*1 is 0.85 or above approximately 5,810 episodes. The average success rate of agents without constraint guidance is 0.85 and above approximately 7,685 episodes. Regarding the number of training episodes required to achieve a success rate of 0.85 and above, the multi-stage constraint is 77% of the single-stage constraint guidance and 58% of the unconstrained guidance. In addition, the maximum average success rate for multi-segment constrained booting is 0.98, the maximum average success rate for single-segment constrained booting is 0.94, and the maximum average success rate for unconstrained booting is 0.98. The results show that using joint Angle constraint to guide the learning of the agent can improve the learning speed without sacrificing the success rate. The results demonstrate the superiority of the proposed framework in the performance of multi-step tasks.

**Figure 8:**
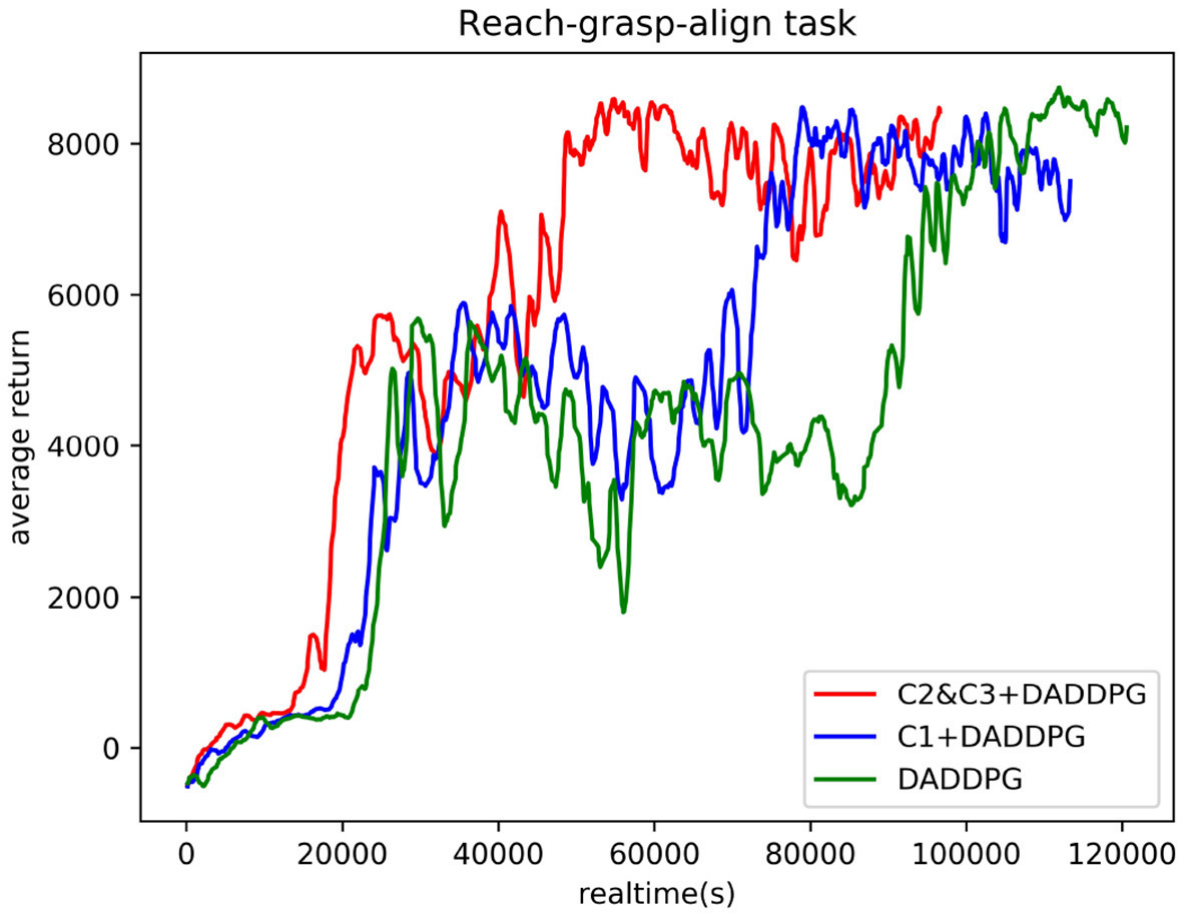
Comparison of actual training time curves of the DADDPG algorithm with single-segment constraint introduction, multi-segment constraint introduction, and no constraint introduction in the reach-grasp-align task of the dual-arm robot.

Figure 8 compares the actual training time of DADDPG algorithm with single-segment constraint introduction, multi-segment constraint introduction, and no constraint introduction in the reach-grasp-align task of the dual-arm robot. The DADDPG algorithm with single-segment constraint introduction, multi-segment constraint introduction, and unconstrained constraint introduction was trained in 10,000 episodes on the same device. As can be observed from the figure, the average cumulative return curve of an agent guided by multi-segment constraints *C*2&*C*3 converges at approximately 48590s, that of an agent guided by single-segment constraints *C*1 converges at approximately 75830s, and that of an agent guided by no constraints converges at approximately 96393s. Regarding actual training time for average cumulative return curve convergence, the training time required for multi-stage constrained guided agent convergence is 64% of that for single-stage constrained guided agent and 50% for unconstrained guided agent. The results show that although the joint angle constraint guidance needs to increase the observation dimension and improve the computational complexity to a certain extent, the actual training time is still significantly reduced. The results further demonstrate the effectiveness of the proposed framework in reducing training time.

## 4 Discussion

Improving the learning efficiency of dual-arm robots' motion planning is always a primary concern, as it can save the investment in time and hardware. In this article, we introduced human joint angle constraints into the DADDPG method for improving the motion planning framework of dual-arm robots. We tested the improved framework on a Baxter dual-arm robot in the Gym simulation environment. The performance of the proposed motion planning framework was evaluated in multi-step tasks (reaching, grasping, and aligning). The results show that the introduced human angle constraints effectively guide robots to learn tasks faster.

Human movement patterns are energy consumption optimal solutions learned through life experience. Inspired by human movement patterns during reaching, grasping, and aligning objects in random places, we extracted human motion features from joint angle curves for faster learning and a higher task completion rate. These features are transformed into constraints in each step during learning. In Section 3.2, three operations were planned using the same constraints. As shown in Figures 6, 8, the real learning time and the number of iterations were reduced. This phenomenon demonstrates that constraining the angle range of the robot joint can narrow the exploration space of the end trajectory, thus improving the learning efficiency. To further enhance learning efficiency, smaller constraints were defined for reaching/grasping and aligning, respectively. The real learning time and the number of iterations were shorter. The effectiveness of the introduced human joint angle constraints is verified.

This study defined the constraints for the joint angle of dual-arm robots. Thus, a smaller search space of the end is obtained. Some other motion parameters, such as joint angular velocity or acceleration, can also be constrained to improve the learning efficiency. The constraints can be defined more strictly according to the human motion features with respect to these motion parameters. Therefore, the proposed constraint-based dual-arm robot motion planning framework has a scalability potential.

In our future studies, the performance of the proposed planning framework will be verified on the objects unseen in the Gym simulation learning. A more complicated task pool will also be developed to show the potential of this work. The additional tasks are mainly focused on three application scenarios: a) dynamic assembly of parts in the factory, b) valve screwing in the space environment, and c) multi-objects sequentially screwing in housekeeping and healthcare.

## 5 Conclusion

This study proposes a motion planning framework based on reinforcement learning guided by human joint angle constraints, and the DADDPG algorithm is selected as part of reinforcement learning. First, the human joint angle is calculated from the demonstration data collected by IMU and mapped to the robot model. The joint angle constraint is extracted piecewise from multiple groups of human demonstrations. Then, the segmented step guidance reward is designed, and the joint angle constraint is introduced into the reinforcement learning algorithm to guide the autonomous learning of the multi-step coordination trajectory of both arms. Finally, for the reach-grasp align task of the two-arm robot, the effectiveness of the proposed framework was verified in terms of training convergence speed, success rate, and training duration under the GYM simulation environment of the Baxter robot. The method will be extended to more complex multi-step tasks and applied to bottle cap screwing scenarios for home services in future studies.

## Data availability statement

The original contributions presented in the study are included in the article/supplementary material, further inquiries can be directed to the corresponding authors.

## Author contributions

KL: Writing – original draft, Writing – review & editing. FZ: Funding acquisition, Resources, Writing – review & editing. WG: Visualization, Writing – review & editing. SL: Writing – review & editing. PW: Formal analysis, Writing – review & editing. LS: Supervision, Writing – review & editing.
